# Elevated expression of Tweety homologue 3 predicts poor clinical outcomes in ovarian cancer

**DOI:** 10.7150/jca.63539

**Published:** 2021-10-20

**Authors:** Junhan Zhou, Yichi Xu, Xin Chen, Fengyun Chen, Jianan Zhang, Xueqiong Zhu

**Affiliations:** 1 Department of Obstetrics and Gynecology, the Second Affiliated Hospital of Wenzhou Medical University, Wenzhou, Zhejiang, 325027, China.; 2 Department of Obstetrics and Gynecology, Taizhou Women and Children's Hospital of Wenzhou Medical University, Taizhou, Zhejiang, 318000, China.

**Keywords:** Tweety homologs, TTYH, ovarian carcinoma, Kaplan-Meier plotter, Oncomine database, prognosis.

## Abstract

**Objective:** To define the alteration of tweety homolog (TTYH) expression in patients with ovarian carcinoma (OC) and its correlation to prognosis.

**Methods:** Kaplan-Meier (KM) plotter was used to evaluate the association between TTYHs expression and clinical outcomes of OC patients. The distribution of 20-year overall survival (OS) and progression-free survival (PFS) was estimated using KM survival plots. The mRNA expression of TTYHs in OC and normal ovarian tissues was confirmed by the Oncomine database. Then, using immunohistochemistry assay, the expression of TTYH1 and TTYH3 proteins in serous OC and normal ovarian tissues was detected. In addition, the protein and mRNA levels of TTYH1 and TTYH3 in human OC cell lines ES-2, A2780 and SKOV3 and normal ovarian epithelial cell lines IOSE80 were assessed by western blotting and real-time quantitative polymerase chain reaction (qRT-PCR).

**Results:** TTYH1 possessed meaningful significance in predicting better prognosis in the serous, advanced stage, and well-differentiated OC patients, while TTYH3 expression predicted worse prognosis in serous, late-stage, and poorly differentiated OC patients. High expression of TTYH1 displayed an association with favorable PFS in OC patients with TP53 mutation. However, enhanced TTYH3 was related to an adverse clinical outcome in TP53-mutated OC patients. In addition, TTYH1 was related to a better clinical outcome in OC patients with platinums-based chemotherapy, but only indicated improved overall survival in OC patients who received taxol or platin + taxol chemotherapy. The up-regulated expression of TTYH3 predicted worse survival in OC patients receiving platin, taxol, or platin + taxol chemotherapy regimen. The levels of TTYH3 mRNA and protein were higher in OC cells and tissues when compared to normal ovarian cells and tissues.

**Conclusions:** TTYH3 was a potential predictor for poor clinical outcome in OC patients, particularly in patients with serous, late-stage, poorly differentiated, TP53-mutation or the patients treated with chemotherapy regimens (platin, taxol, or platin + taxol).

## Background

As one of the most common gynecologic malignancies, ovarian carcinoma (OC) possesses the highest lethality in female globally 1. There are approximately 313,959 new diagnosed OC cases and 207,252 new deaths per year worldwide based on statistics 2. Due to the nonspecific symptoms as well as the lack of effective prognostic biomarkers in early lesions, the prognosis in late-stage OC patients postoperatively is generally unfavorable with the five-year OS below 30% 3. The discovery of novel markers for precisely predicting the prognosis is urgently needed for OC patients.

The tweety homologs form a large gene family that encode evolutionarily typical conserved transmembrane chloride channels with large conductance, including tweety homolog 1 (TTYH1), tweety homolog 2 (TTYH2), and tweety homolog 3 (TTYH3) 4-6. TTYH1 is reciprocally regulated by volume swelling and activation of chloride channels, which is restricted to nerve tissue primarily with an unclear role in human neuronal physiology. TTYH2 and TTYH3 function as calcium-activated chloride channels extensively regulating cellular activity 7, 8. Upregulation of TTYH1 has been validated in various cancers, including glioma, astrocytoma, and other cancers. TTYH1 is involved in cell-cell communication, adhesion, and migration in mammalian neuronal as a transmembrane receptor 9-11. Additionally, the pediatric brain tumors are developed under the control of TTYH1-promotor integrated with microRNA cluster C19MC 12. Overexpression of TTYH2 induces proliferation and migration, particularly in colorectal cancer 13. Besides, small interference RNA (siRNA)-mediated TTYH2 gene silencing results in the marked reduction in invasion and migration of osteosarcoma cells 14. To the best of our knowledge, TTYH3 is mainly expressed in the calcium-dependent excitatory tissues particularly in the brain, heart, and skeletal muscle 4. However, studies regarding TTYH3 have almost exclusively focused on its biochemical structure rather than prognosis prediction or its expression pattern in cancers.

Therefore, this study aimed to evaluate prognostic roles of TTYHs and the expression patterns in OC. The aberrant expression of TTYHs was commonly observed in OC based on publicly available database and clinical information. TTYHs might have the impact on the prognosis of OC patients.

## Materials and Methods

### Kaplan-Meier (KM) Plotter Database

Analyses on the relationship between mRNA expression of TTYHs and prognostic values in OC patients were performed using survival curves, which was acquired from KM Plotter (http://kmplot.com/analysis/), a searchable, accessible for free, and integrated online database containing clinical information and gene expression for breast, lung, gastric, and ovarian cancers. TTYH genes (TTYH1, TTYH2, and TTYH3) were individually uploaded into the database, in which the samples of OC patients were categorized into two parts (low or excessive expression) in accordance to the best cut-off of TTYHs mRNA expression values, and the distribution of 20-year overall survival (OS) and progression-free survival (PFS) were estimated using KM survival plots. The hazard ratios (HRs), 95% confidence intervals (CIs), and log-rank *P*-values were computed in terms of histological subtypes, stages, grades, TP53 mutation status, and chemotherapy regimen for OC patients, considering *P* value less than 0.05 to be significant.

### Oncomine Database

The Oncomine database (https://www.oncomine.org/), a publicly available cancer microarray database, was employed to make further identification in matched normal ovary and OC tissues on mRNA levels of TTYHs, by which the transcriptional alteration in different types of cancers and corresponding normal tissues could be compared directly. A total of 5 normal ovary samples and 20 ovarian serous adenocarcinoma tissues were obtained from the database. *P*-value less than 0.05, fold-changes more than 2, and gene rank in top 10% were selected respectively as the cut-off threshold for significantly differential expression with all data presented as box plots.

### Tissue samples

Surgical tissue specimens obtained from patients who were operated in the Second Affiliated Hospital of Wenzhou Medical University from 2014 to 2018 with normal ovary or serous OC were applied for immunohistochemistry. None of the serous OC patients had received any antitumor treatment pre-operatively. Besides, the patients with only unilateral benign ovarian lesions who underwent bilateral ovariectomy were enrolled into our study as the control group. Fourteen serous ovarian cancer specimens from patients (median age 51 years, 34-74 years) selected for the analysis of TTYH1 and TTYH3 were all confirmed post-operatively by histopathological examination. And the control group of TTYH1 and TTYH3, containing 12 cases of normal ovarian tissue samples which were confirmed post-operatively by histopathological examination, was retrieved from patients aged from 45 to 72 (median age, 55 years) who underwent bilateral ovariectomy due to unilateral ovarian lesions.

The protocol of this research was reviewed and approved by the Ethics Committee of the Second Affiliated Hospital of Wenzhou Medical University. Written informed consent was provided by all enrolled subjects or their relatives prior to participation.

### Immunohistochemistry

Tissues were formalin-fixed, paraffin-embedded, and sectioned at 4 μm. Slices of paraffin-embedded samples were heated at 65°C for 2 h before dewaxing in xylene and rehydration using a graded series of ethanol. Slices were processed for antigen extraction, and then the slices were added with 0.3% hydrogen peroxide in methanol on purpose of inhibiting the endogenous peroxidase activity. Subsequently, free-floating sectional tissue samples blocking with 5% normal goat serum were then soaked in 1:200 rabbit anti-human TTYH1 antibody or 1:50 rabbit anti-human. TTYH3 antibody were added for incubation overnight at temperature adjusted to 4 °C following with binding of secondary antibody (biotinylated goat anti-rabbit) for 1 h. 3'3-diaminobenzidine tetrahydrochloride (DAB) was added to slices as a chromogenic agent following with hematoxylin counterstaining. A total of 100 cells were counted by randomly choosing 10 high-magnification fields of vision. The staining extent was scored ground on the proportion of cells with positive staining as follows: 0-5% were scored as 0, 6-24% were scored as 1 point, 25-49% were scored as 2 points, 50-74% were scored as 3 points, 75-100% were scored as 4 points. The scoring criteria for immunostaining intensity were distributed as follows: no staining was scored as 0, faint yellow was scored as 1 point, yellowish-brown was scored as 2 points, and brown was scored as 3 points. The product of positively stained cells ratio and intensity of staining was defined as the protein expression of TTYH1 and TTYH3. Meanwhile, negative and positive controls were set in each staining run within this experiment.

### Cell lines

IOSE80 (normal ovarian epithelial cell lines), ES-2 (human ovarian clear cell carcinoma cell lines), A2780 and SKOV3 cells (human epithelial ovarian carcinoma cell lines) were bought from Cell Bank of Chinese Academy of Sciences (Shanghai, China). Cells were maintained in RPMI 1640 medium (Biochrom AG, Berlin, Germany) added with 10% fetal bovine serum (FBS, Gibco, USA), and 1% penicillin, and streptomycin (Thermo Scientific, USA) at temperature of 37 °C in 5% CO_2_ conditions_._

### Real-time quantitative polymerase chain reaction (qRT-PCR)

When the cultured cells grew to 70% to 80% confluence in cell culture plates, the mediums were removed and cells were washed using phosphate buffered saline (PBS), and then cells were trypsinized with 0.25% Trypsin-ethylene diamine tetraacetic acid (EDTA) (Sigma, USA). TRlzol reagent was applied for extraction of total RNA from all cells, and then the amount and purity of RNA were evaluated through the Nanodrop 2000 ultraviolet spectrophotometer. Reverse transcript mRNA into cDNA and the obtained template cDNA were subjected to real-time quantitative PCR. The primer sequences were as follows: TTYH1 forward, 5'-TGGCGAAGCAGAGCAAG-3' and antisense 5'-AGGGTCTGGATTGGAGCA-3'; TTYH3 forward, 5'-CAG AGT GGG GAG GGG AGT-3' and antisense 5'-CTG GGC AGG TTG GCT GT-3'; and GAPDH forward, 3'-ACC CAG AAG ACT GTG GAT GG-5' and antisense, 3'-TCT AGA CGG CAG GTC AGG TC-5'. PCR reactions were conducted on the Exicycler™ 96 fluorescence quantitative instrument (Bioneer, Daejeon, Korea). The amount of each gene mRNA value was normalized to the value of GAPDH mRNA and the relative mRNA abundance for the surveyed samples was estimated by using the 2^-ΔΔCt^ method. At least three times repetitions were performed independently during this experiment.

### Western blot analysis

Radioimmunoprecipitation assay (RIPA) lysis buffer added with proteinase inhibitor was used for protein isolation. The cell lysates were cleared by centrifugation and then the supernatants were collected, bicinchoninic acid (BCA) assay was conducted on the concentration of proteins. Total proteins (10 μg) were loaded per lane and isolated via 10% sodium dodecyl sulfate-polyacrylamide gel electrophoresis (SDS-PAGE) following with transfer to the polyvinylidene fluoride (PVDF) membranes. Following sealing with 5% skim milk powder solution at room temperature for 1 h, the membranes were stored overnight at temperature of 4°C. Primary antibodies applied for western blot contained: 1:1000 anti-TTYH1 polyclonal rabbit antibody (proteintech, 26973-1-AP) and 1:1000anti-TTYH3 polyclonal rabbit antibody (Abcam, ab240580) and 1:5000 anti-GAPDH monoclonal rabbit antibody (proteintech, 60004-1-lg). After washing the membranes for three times, the membranes were soaked in the secondary antibody for 45 min followed by visualizing with electrochemiluminescence (ECL) reagent (Thermo Fisher Scientific), exposure, and digital imaging. At least three times repetitions were carried out independently during all experiments.

### Statistical analysis

The association among TTYH expression, different clinicopathologic factors, and patient survival was analyzed by using the KM. The comparisons between subgroups were conducted by the log-rank analysis. SPSS software 22.0 (SPSS Inc., Chicago, IL, USA) and GraphpadPrism 8.0 (GraphPad Software, U.S.A.) were conducted on statistical analyses. Normally distributed data were presented as mean values ± standard deviation (SD) and employed for validating significance by using Student's t-test, whereas non-normally distributed data were applied by using Mann-Whitney U-test, considering *P* less than 0.05 as the standard of statistical significance.

## Results

### TTYH2 mRNA expression is not associated with all OC patients' survival

**Figure 1:**
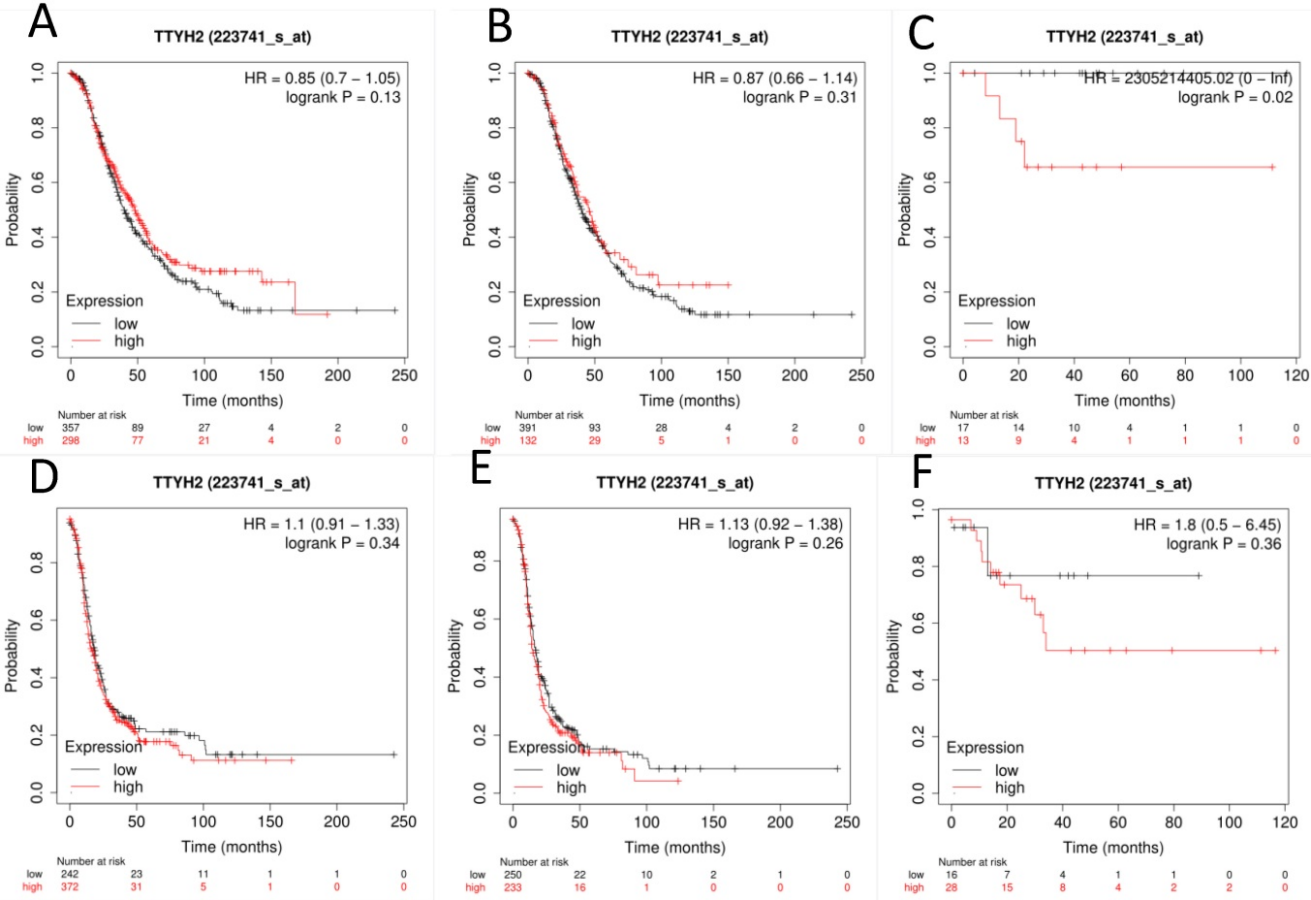
**The prognostic significance of TTYH2 in OC patients.** Curves of OS were drawn for (A) all OC patients (N=655), (B) patients with serous OC (N=523), (C) patients with endometrioid OC (N=30); Curves of PFS were drawn for (D) all OC patients (N=614), (E) patients with serous OC (N=483), (F) patients with endometrioid OC (N=44).

The prognostic value of TTYH2 (Affymetrix ID: 223741_s_at, Fig. 1) was preliminarily analyzed by generating survival curves in the database. High mRNA expression of TTYH2 was only discovered to be potentially linked to poor OS in patients with endometrioid OC (HR, 2305214405.02; 95% CI, 0-lnf; *P* =0.02), but not in all OC or serous OC patients. Similar in PFS, TTYH2 mRNA expression above or below best cut-off did not show any differences in all OC patients, patients with endometrioid OC, or patients with serous OC.

### High expression of TTYH1 and TTYH3 mRNA is correlated with survival of patients with OC

**Figure 2:**
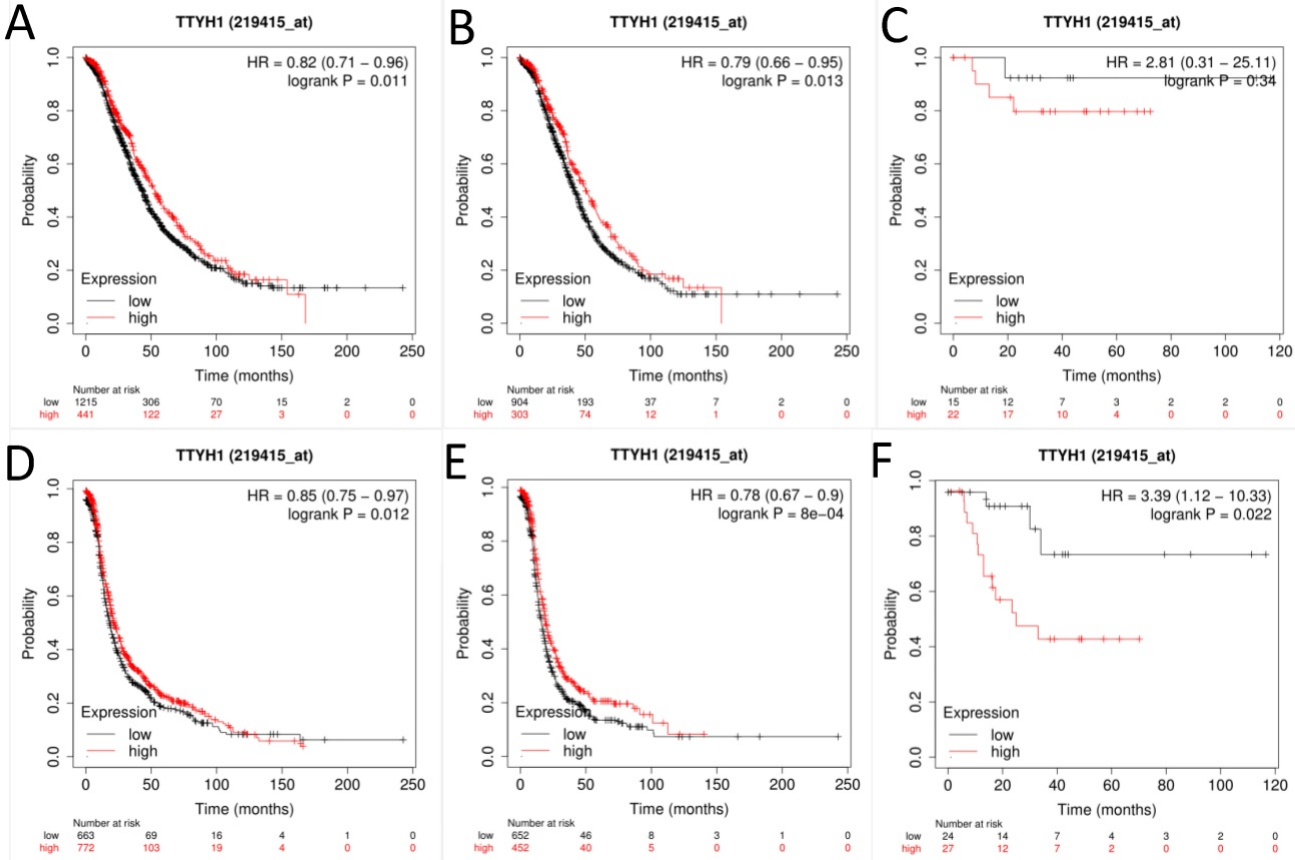
**The prognostic significance of TTYH1 in OC patients.** Curves of OS were drawn for (A) all OC patients (N=1656), (B) patients with serous OC (N=1207), (C) patients with endometrioid OC (N=37); Curves of PFS were drawn for (D) all OC patients (N=1435), (E) patients with serous OC (N=1104), (F) patients with endometrioid OC (N=51).

Next, the prognostic significance of TTYH1 (Affymetrix ID: 219415_at, Fig. 2) was comprehensively assessed. The curves showed that up-regulated TTYH1 mRNA expression indicated better OS and PFS in all patients with OC (HR, 0.82; 95% CI, 0.71-0.96; *P* =0.011; HR, 0.79; 95% CI, 0.66-0.95; *P* =0.013, respectively) as well as patients with serous OC (HR, 0.85; 95% CI, 0.75-0.97; *P* =0.012; and HR, 0.79; 95% CI, 0.67-0.9; *P* =0.0008, respectively). In patients with endometrioid OC, although high expression of TTYH1 mRNA exhibited significant correlation with favorable PFS, the expression of TTYH1 had no significant relationship with OS (HR, 3.39; 95% CI, 1.12-10.33; *P* =0.022; and HR, 2.81; 95% CI, 0.31-25.11; *P* =0.37, respectively).

**Figure 3:**
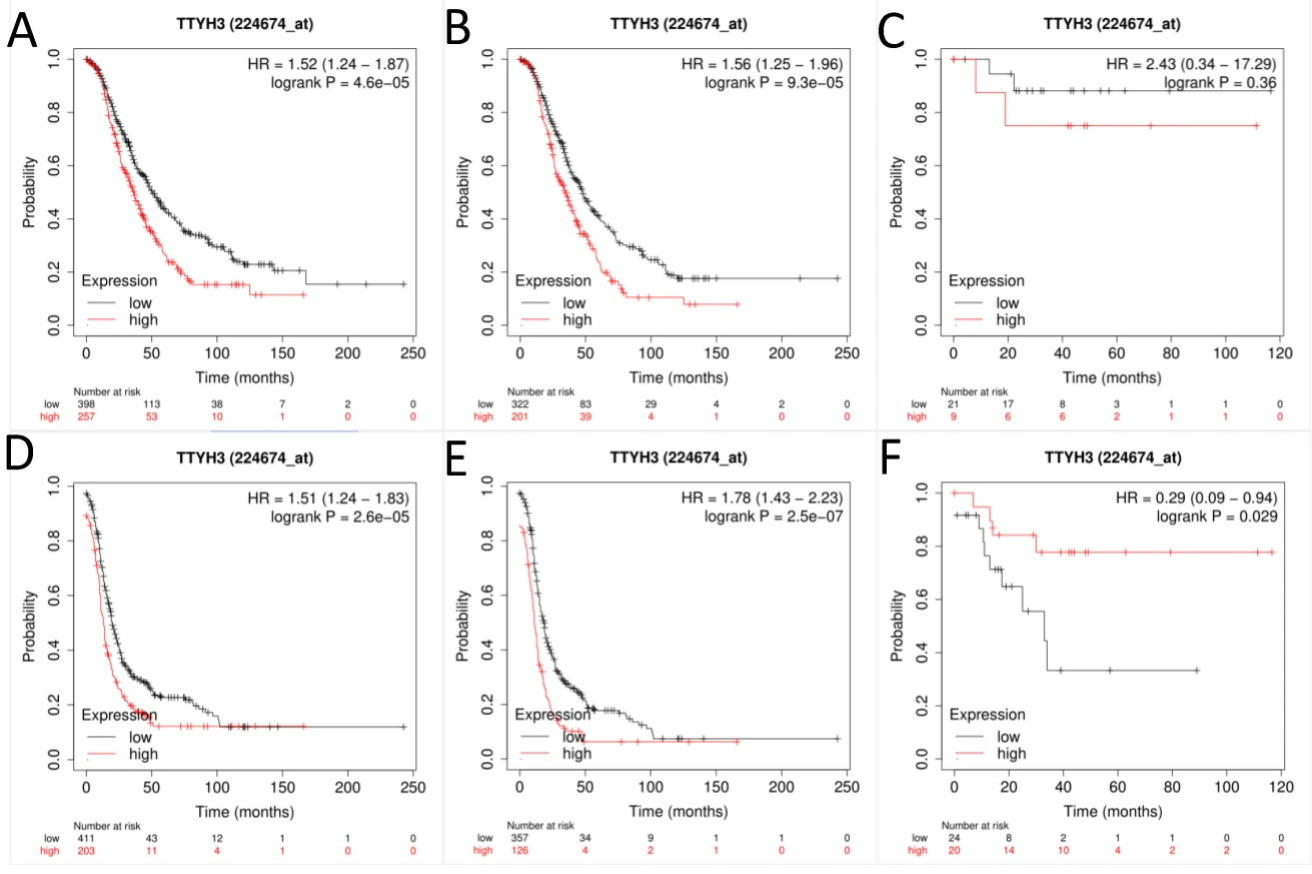
**The prognostic significance of TTYH3 in OC patients.** Curves of OS were drawn for (A) all OC patients (N=655), (B) patients with serous OC (N=523), (C) patients with endometrioid OC (N=30); Curves of PFS were drawn for (D) all OC patients (N=614), (E) patients with serous OC (N=483), (F) patients with endometrioid OC (N=44).

Next, the prognostic effect of TTYH3 mRNA expression was examined (Affymetrix ID: 224674_at, Fig. 3). Elevated TTYH3 mRNA expression showed significantly shorter OS and PFS both in all patients with OC (HR, 1.52; 95% CI, 1.24-1.87; *P* =0.0000; and HR, 1.56; 95% CI, 1.25-1.96; *P* =0.0001, respectively) and patients with serous OC (HR, 1.51; 95% CI, 1.24-1.83; *P* =0.0000; and HR, 1.78; 95% CI, 1.43-2.23; *P* =0.0000, respectively). However, excessive TTYH3 mRNA expression was connected with better PFS but not to OS regard to patients with endometrioid OC (HR, 0.29; 95% CI, 0.09-0.94; *P* =0.029; and HR, 2.43; 95% CI, 0.34-17.29; *P* =0.36, respectively).

### Relationship between TTYH1 and TTYH3 mRNA expression and other clinicopathologic characteristics of OC patients

Whether the mRNA expression levels of TTYH1 and TTYH3 had a relationship with clinicopathologic characteristics in OC patients, including stages, grades, TP53 mutation status and chemotherapy, further investigation of this relationship was conducted subsequently. Up-regulated TTYH1 mRNA expression indicated favorable OS and PFS in stages III and IV patients with OC, while the TTYH1 mRNA expression was observed to have a relationship with adverse PFS in stage I, and II shown in Table 1. Moreover, up-regulated mRNA expression of TTYH3 was correlated with unfavorable OS in overall carcinoma stages while only predicted unfavorable PFS in stage III, and IV.

Increased TTYH1 mRNA expression showed a significant correlation with favorable OS in grade I and better PFS in grade I and III, which was shown in Table 2. Meanwhile, the data revealed that up-regulated mRNA expression of TTYH3 displayed a significant correlation with worse OS and PFS in patients with OC in grade III.

As shown in Table 3, a comparison between TTYH1 mRNA expression and TP53 mutation status suggested that increased TTYH1 mRNA expression predicted better PFS in OC patients with TP53 mutation. Contrary to TTYH1, elevated mRNA expression of TTYH3 suggested unfavorable OS and PFS in TP53-mutated OC patients. However, augmented TTYH3 mRNA expression indicated a relation to favorable OS in TP53 wild-type OC patients.

Interestingly, enhanced TTYH1 mRNA expression indicated a direct correlation with favorable OS in OC patients receiving platin, taxol, or platin + taxol chemotherapy scheme. Additionally, elevated TTYH1 mRNA expression was confirmed to have a relationship with favorable PFS in patients treated with platin. It was revealed that the augmentation of TTYH3 mRNA expression was verified to be related to adverse OS and PFS in OC patients under the treatment of platin, taxol, or platin + taxol (Table 4).

### Comparisons of TTYHs mRNA transcription levels and protein levels between OC tissues and normal ovarian tissues

**Figure 4:**
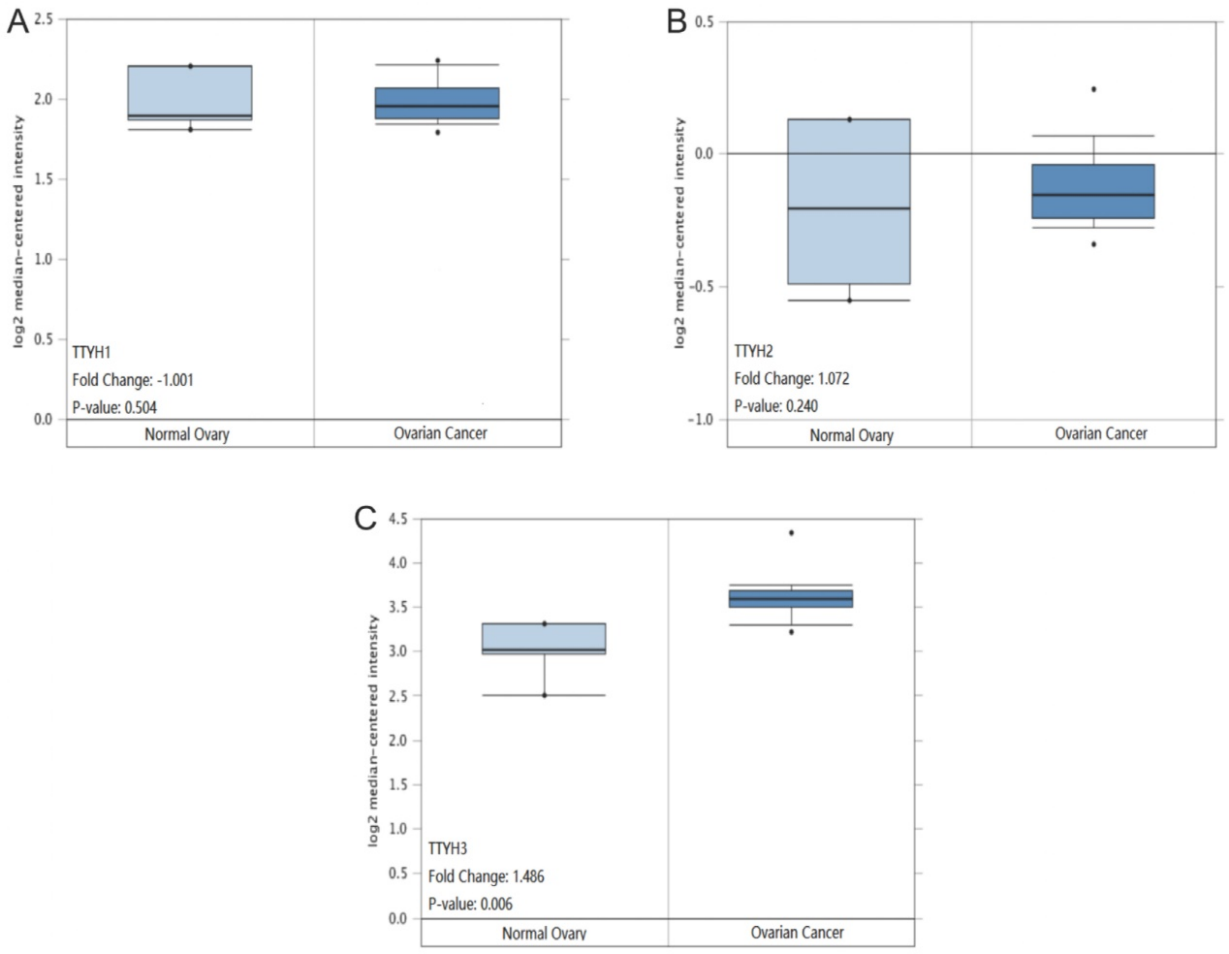
** TTYHs mRNA expression analysis in OC tissues and normal ovary tissues.** (A) TTYH1, (B) TTYH2, (C) TTYH3.

TTYH1 and TTYH2 mRNA transcription levels displayed no significant differences in OC tissues compared to normal counterparts retrieved from the Oncomine database (*P* =0.504, Fig. 4A; *P* =0.240, Fig. 4B). However, the TTYH3 mRNA expression data in OC tissues was revealed to be obviously higher in comparison with normal ovarian tissues (*P*=0.006, fold-changes=1.486. Fig. 4C).

**Figure 5:**
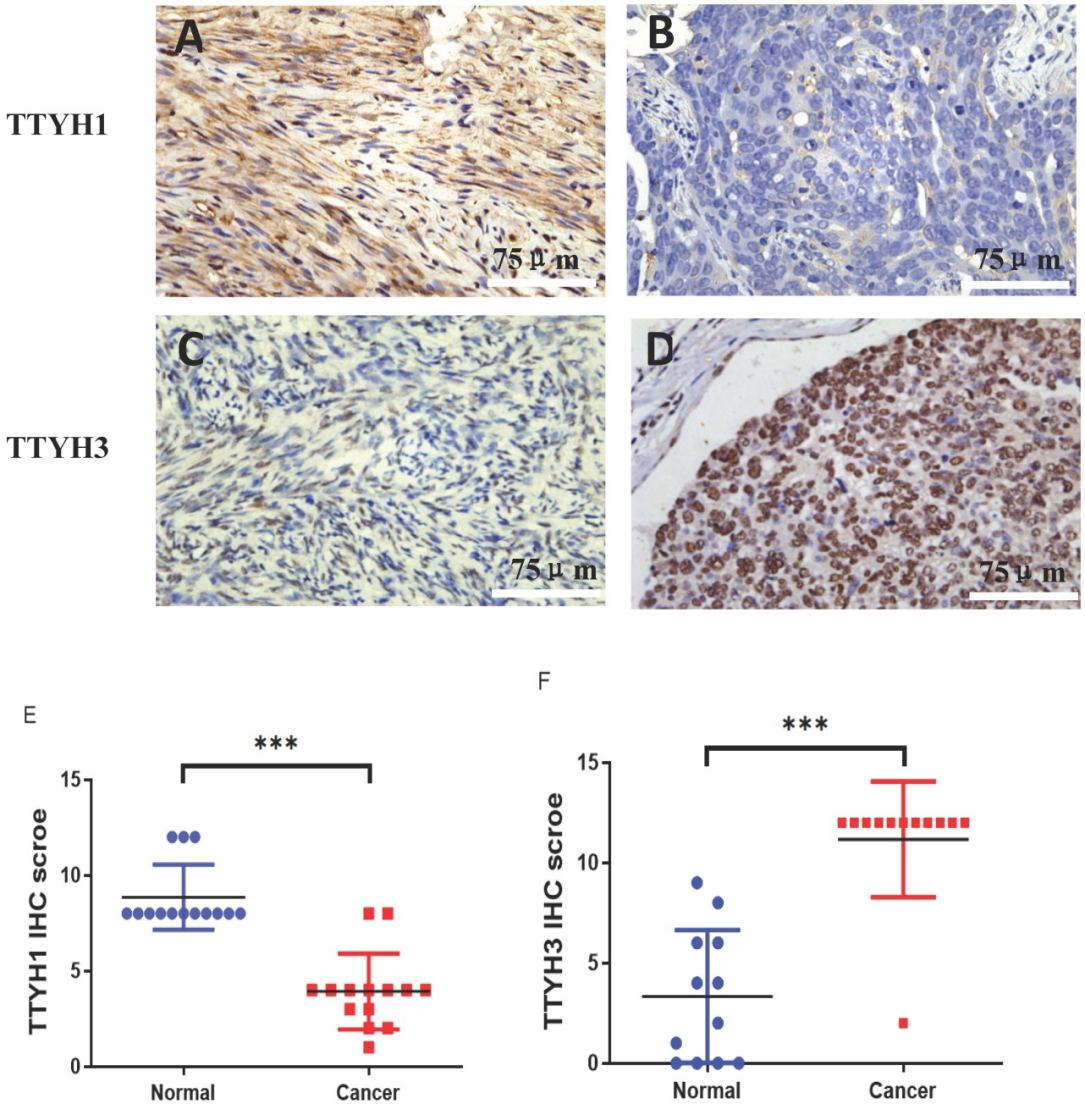
** TTYH1 and TTYH3 protein expression in OC tissues and normal ovarian tissues by immunohistochemistry.** Immunohistochemical staining of TTYH1 in (A) normal ovarian tissues, (B) OC tissues, and (E) their different expression. Immunohistochemical staining of TTYH3 in (C) normal ovarian tissues, (D) OC tissues and (F) their different expression. Scale bar= 75 μm.

The differential protein expression of TTYH1 and TTYH3 between OC tissue samples and normal ovarian tissue samples was detected by immunohistochemistry. Positive protein expression of TTYH1 in normal ovarian tissues (8.86 ± 1.70) was significantly higher than those in OC tissues (3.93 ± 1.98) (*P* <0.001; Fig. 5A, B, and E). TTYH3 expression was rarely detectable in normal ovarian tissues (3.30 ± 3.12) but was greatly higher in OC tissues (11.17 ± 2.88) with a statistical difference (*P* <0.001; Fig. 5 C, D, and F).

### Expression level of TTYH1 and TTYH3 in normal ovarian and OC cell lines

**Figure 6:**
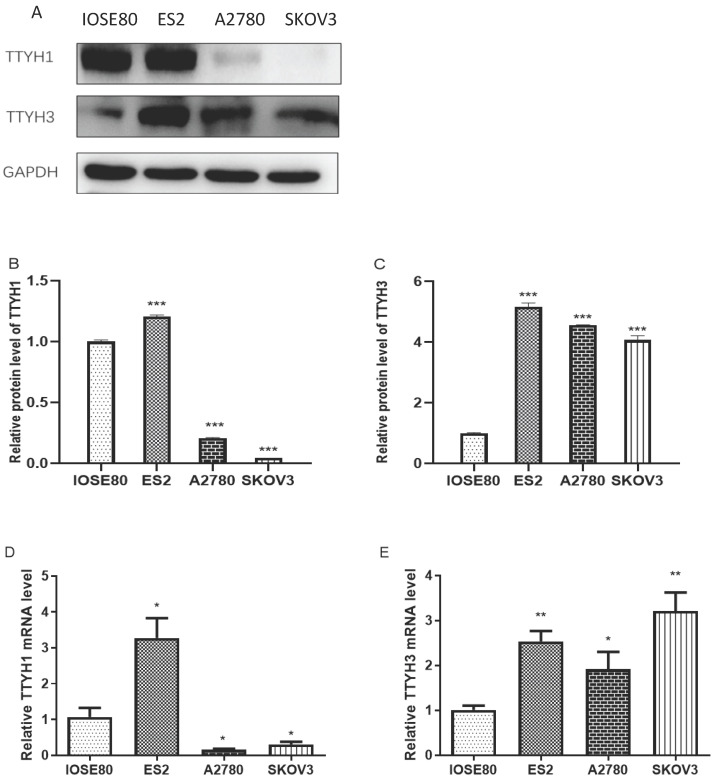
** TTYH1 and TTYH3 expression in normal ovarian cell line and OC cell lines.** (A-C) Western blot was conducted to detect the protein levels of TTYH1 and TTYH3, and the protein values of GAPDH were used as an internal control. (D-E) qRT-PCR was conducted to detect the mRNA values of TTYH1 and TTYH3, and the mRNA values of GAPDH were used as an internal control. The data are presented as mean values ± SD,*: compared with IOSE80 group, ^*^P<0.05, ^**^P<0.01, ^***^P<0.001.

The mRNA and protein expression of TTYH1 was obviously augmented in ES2 cells in comparison with IOSE80 cells, whereas the TTYH1 mRNA and protein expression levels in SKOV3 and A2780 cells were significantly lower compared with IOSE80 cells (*P* <0.05). As shown in Fig. 6, up-regulated TTYH3 expression at mRNA and protein expression levels was detected in all types of OC cell lines (ES2, SKOV3, A2780) in comparison with those in IOSE80 (*P*<0.05).

## Discussion

To examine the prognostic values of TTYHs in OC patients, we attempted to systematically investigate the expression pattern and TTYHs-related survival in OC patients. Among OC patients, TTYH1 predicted better clinical outcomes while TTYH3 predicted worse prognosis. TTYH2 presented no significant association with outcomes in OC patients. Hence, further analysis among TTYH1 and TTYH3 expression, clinicopathological features, and the outcomes of OC patients were comprehensively studied. Besides, the data retrieved from the Oncomine database or the results obtained from qRT-PCR, western blot, immunohistochemistry analyses, higher levels of TTYH3 were detected in OC specimens in comparison to non-cancer normal ovarian specimens.

TTYH2 gene is located on chromosome band 17q24 15. Expression of TTYH2 has been found in various organs, including the ovary, heart, brain, spleen, and peripheral blood leukocytes 16. Furthermore, a high expression level of TTYH2 was found in muscle-invasive bladder carcinomas, adrenal cortical neoplasms, and brain metastases of solid tumors 13, 17. In addition, the upregulation of TTYH2 expression was detected in renal cell cancer, suggesting that its upregulation might play an oncogenic role in renal tumorigenesis 16. In contrast to normal colon tissues, TTYH2 expression was significantly up-regulated in colon cancer tissues 18. Silence of TTYH2 by transfecting siRNA markedly inhibited proliferation and migration of colon cancer cells 13. However, our study demonstrated that the high expression of TTYH2 seemed irrelevant to the clinical outcomes of OC patients. Therefore, the prognostic value of TTYH2 in OC patients is limited.

TTYH1 is a membrane protein predominantly restricted to the neural tissue among normal tissues 19. TTYH1 plays an essential role in normal brain development and serves as a potent regulator of cell multiplication 20. A recent study has indicated that silencing of TTYH1 inhibited the progression of glioma 19. In the present study, TTYH1 predicted an improved prognosis in all OC patients, particularly in patients with serous OC. Furthermore, elevated TTYH1 expression had a favorable prognosis to grade I, stage III and IV. This suggested that TTYH1 possessed meaningful significance in predicting better prognosis in the well-differentiated, advanced stage, and serous OC patients. Moreover, the transcription and protein levels of TTYH1 in OC cells were remarkably lower in human epithelial ovarian carcinoma cell lines A2789 and SKOV3 in comparison with normal ovarian cells. TTYH1 expression levels in human ES-2 ovarian clear cell carcinoma cell line were greatly higher in comparison with normal ovarian cells. Considering the inconsistent expression values of TTYH1 in OC cells, TTYH1 might exert distinct roles in different histological types of OC. TTYH1 was correlated to favorable prognosis with epithelial ovarian carcinoma, particularly in serous ovarian carcinoma, whereas the roles of TTYH1 in ovarian clear cell carcinoma or other kinds of OC remained unclear. Evaluation of the TTYH1 expression levels between normal ovarian tissues and OC tissues extracted from fresh specimens of patients were needed for further clinical studies.

To date, reports of TTYH3 in malignant carcinomas and the relationship with prognosis are limited. The role of TTYH3 in the pathogenesis of tumors remains also ambiguous. An analysis confirmed that the expression of TTYH3 was obviously higher in gastric carcinoma than those in normal tissues, and the upregulation of TTYH3 expression in gastric cancer patients reflected worse prognostic to a certain extent 14. In our study, TTYH3 expression indicated worse clinical outcomes in all OC patients, particularly in serous OC patients. Moreover, enhanced TTYH3 expression was found to be related to the adverse prognosis in grade III, stage III and IV. This implied that elevated TTYH3 expression predicted worse prognosis in late-stage, poorly differentiated OC patients. Consistent to the expression pattern of TTYH3 in gastric cancer, TTYH3 presented higher expression in OC cells and tissues in mRNA or protein expression levels compared to normal ovarian cells and tissues. It was explored that the enhanced TTYH3 expression at the mRNA level was consistent with that at the protein level, implying that TTYH3 expression at both transcriptional and translational levels were relevant to unfavorable prognosis in OC patients. The TTYH3-related signaling pathways involved in up-regulated expression in OC cells were poorly understood and should be clarified in further investigations.

The prognostic significance of TTYH1 and TTYH3 in TP53-mutated cancers remains unclear; the available literature is rare to date. Our data analysis investigated enhanced TTYH3 expression was related to worse prognosis in TP53-mutated OC patients. However, up-regulated expression of TTYH1 had an association with favorable PFS in OC patients with TP53 mutation. We therefore speculated that the binding of TTYH1 and TTYH3 to the promoter region of TP53 regulated TP53-mediated tumorigenesis, and the expression of TTYH1 and TTYH3 could be considered as biomarkers of prognosis in TP53-mutated OC patients. Furthermore, high expression level of TTYH3 predicted unfavorable survival rate in OC patients under the treatment of platin, taxol, or platin + taxol chemotherapy scheme. Meanwhile, TTYH1 was found to be associated with a better prognosis in OC patients receiving chemotherapy scheme with platin, taxol, or platin + taxol. The results of the present study imply that TTYH1 and TTYH3 might be potent predictors of prognosis in OC patients treated with anticancer chemotherapy.

## Conclusion

TTYH1 was a favorable prognostic biomarker of for OC patients. However, the mRNA expression of TTYH1 between OC and normal tissues was inconsistent, which provided weak support. Therefore, the value of TTYH1 to predict the prognosis of OC needs further verification. Increased TTYH3 expression might be an effective predictor of poor prognosis for OC, especially in poorly differentiated, late-stage, and serous OC patients. TTYH3 indicated a poor prognosis in OC patients with TP53 mutation and the patients who received chemotherapy with platins, taxol, or platin + taxol. This comprehensive analysis raises the value of TTYH3 in predicting the prognosis of OC patients. These results would lead to the development of innovative predictor for future OC targeted therapy.

## Figures and Tables

**Table 1:** The relationship between TTYHs-related prognosis and clinical stages in patients with OC.

TTYH subtypes	Clinical stages		OS			PFS	
		Cases	HR (95%CI)	*P-*value	Cases	HR (95% CI)	*P-*value
TTYH1	Ⅰ+Ⅱ	135	1.6 (0.66-3.86)	0.3	163	2.32 (1.08-4.96)	0.026*
	Ⅲ+Ⅳ	1220	0.79 (0.67-0.92)	0.0025*	1081	0.77 (0.67-0.89)	0.0004*
TTYH2	Ⅰ+Ⅱ	83	0.33 (0.07-1.46)	0.12	115	0.49 (0.22-1.09)	0.075
	Ⅲ+Ⅳ	487	0.81 (0.62-1.05)	0.11	494	1.1 (0.9-1.33)	0.36
TTYH3	Ⅰ+Ⅱ	83	3.6 (1.29-10.05)	0.0092*	115	1.82 (0.88-3.75)	0.1
	Ⅲ+Ⅳ	487	1.57 (1.25-1.97)	0.0001*	1081	1.56 (1.27-1.93)	0.0000*

*P<0.05.

**Table 2:** The Relationship between TTYHs-related prognosis and pathological grades in patients with OC.

TTYH subtypes	pathological grade		OS			PFS	
		Cases	HR (95%CI)	*P-*value	Cases	HR (95% CI)	*P-*value
TTYH1	Ⅰ	56	0.2 (0.06-0.7)	0.0053*	37	0.24 (0.08-0.75)	0.0078*
	Ⅱ	324	0.76 (0.55-1.06)	0.11	256	1.21 (0.9-1.62)	0.21
	Ⅲ	1015	0.9 (0.75-1.08)	0.24	837	0.8 (0.68-0.95)	0.01*
TTYH2	Ⅰ	41	3.09 (1.03-9.29)	0.035*	28	2.41 (0.6-9.65)	0.2
	Ⅱ	162	0.8 (0.5-1.27)	0.34	161	0.83 (0.57-1.2)	0.31
	Ⅲ	392	0.91 (0.69-1.19)	0.49	315	0.84 (0.63-1.11)	0.22
TTYH3	Ⅰ	41	2.42 (0.75-7.78)	0.13	28	2.89 (0.36-23.15)	0.29
	Ⅱ	162	1.52 (0.95-2.43)	0.081	161	0.71 (0.49-1.02)	0.064
	Ⅲ	392	1.49 (1.15-1.95)	0.0028*	315	1.84 (1.42-2.39)	0.0000*

Pathological grade: grade I represented high differentiation; grade Ⅱ represented moderate differentiation; grade Ⅲ represented poor differentiation. *P<0.05.

**Table 3:** The Relationship between TTYHs-related prognosis and TP53-mutated status in patients with OC.

TTYH subtypes	TP53 mutation		OS			PFS	
		Cases	HR (95%CI)	*P-*value	Cases	HR (95%CI)	*P-*value
TTYH1	Yes	506	0.77 (0.58-1.01)	0.059	483	0.72 (0.57-0.9)	0.0046*
	NO	94	0.6 (0.35-1.03)	0.061	84	0.64 (0.38-1.08)	0.089
TTYH2	Yes	124	0.76 (0.52-1.12)	0.17	124	0.77 (0.52-1.13)	0.18
	NO	19	1.59 (0.55-4.56)	0.38	19	0.65 (0.25-1.72)	0.38
TTYH3	Yes	124	1.64 (1.1-2.43)	0.014*	124	2.48 (1.69-3.65)	0.0000*
	NO	19	0.31 (0.1-0.96)	0.032*	19	0.41 (0.13-1.34)	0.13

*P<0.05.

**Table 4:** The Relationship between TTYHs-related prognosis and chemotherapy strategies in patients with OC.

TTYH subtypes	Chemother-apy		OS			PFS	
		Cases	HR (95%CI)	*P-*value	Cases	HR (95%CI)	*P-*value
TTYH1	platin	1409	0.82 (0.7-0.95)	0.0084*	1259	0.84 (0.74-0.96)	0.0079*
	taxol	793	0.79 (0.65-0.95)	0.015*	715	0.85 (0.72-1.01)	0.069
	platin+taxol	776	0.77 (0.64-0.94)	0.0095*	698	0.86 (0.72-1.03)	0.095
TTYH2	platin	478	1.11 (0.87-1.4)	0.4	502	1.13 (0.93-1.37)	0.22
	taxol	357	0.86 (0.64-1.16)	0.33	381	1.18 (0.94-1.48)	0.16
	platin+taxol	356	0.87 (0.64-1.17)	0.35	380	1.17 (0.93-1.47)	0.17
TTYH3	platin	478	1.57 (1.24-1.98)	0.0002*	502	1.51 (1.22-1.87)	0.0001*
	taxol	357	1.55 (1.16-2.07)	0.0026*	381	1.37 (1.08-1.73)	0.0085*
	platin+taxol	356	1.54 (1.16-2.06)	0.0028*	380	1.37 (1.09-1.74)	0.0076*

*P<0.05.

## Abbreviations

TTYH: Tweety homolog
OC: Ovarian carcinoma
KM: Kaplan-Meier
OS: Overall survival
PFS: Progression-free survival
qRT-PCR: Real-time quantitative polymerase chain reaction
HR: hazard ratio
CI: Confidence interval
PBS: Phosphate-buffered saline
EDTA: Ethylene diamine tetraacetic acid
RIPA: Radioimmunoprecipitation assay
BCA: Bicinchoninic acid
SDS-PAGE: Sodium dodecyl sulfate-polyacrylamide gel electrophoresis
PVDF: Polyvinylidene fluoride
ECL: Electrochemiluminescence
SD: Standard deviation
